# Primary Care Practices’ Ability to Report Electronic Clinical Quality Measures in the EvidenceNOW Southwest Initiative to Improve Heart Health

**DOI:** 10.1001/jamanetworkopen.2019.8569

**Published:** 2019-08-07

**Authors:** Kyle E. Knierim, Tristen L. Hall, L. Miriam Dickinson, Donald E. Nease, Dionisia R. de la Cerda, Douglas Fernald, Molly J. Bleecker, Robert L. Rhyne, W. Perry Dickinson

**Affiliations:** 1 University of Colorado School of Medicine, Department of Family Medicine, Aurora; 2 University of New Mexico School of Medicine, Department of Family and Community Medicine, Albuquerque

## Abstract

**Importance:**

The capability and capacity of primary care practices to report electronic clinical quality measures (eCQMs) are questionable.

**Objective:**

To determine how quickly primary care practices can report eCQMs and the practice characteristics associated with faster reporting.

**Design, Setting, and Participants:**

This quality improvement study examined an initiative (EvidenceNOW Southwest) to enhance primary care practices’ ability to adopt evidence-based cardiovascular care approaches: aspirin prescribing, blood pressure control, cholesterol management, and smoking cessation (ABCS). A total of 211 primary care practices in Colorado and New Mexico participating in EvidenceNOW Southwest between February 2015 and December 2017 were included.

**Interventions:**

Practices were instructed on eCQM specifications that could be produced by an electronic health record, a registry, or a third-party platform. Practices received 9 months of support from a practice facilitator, a clinical health information technology advisor, and the research team. Practices were instructed to report their baseline ABCS eCQMs as soon as possible.

**Main Outcomes and Measures:**

The main outcome was time to report the ABCS eCQMs. Cox proportional hazards models were used to examine practice characteristics associated with time to reporting.

**Results:**

Practices were predominantly clinician owned (48%) and in urban or suburban areas (71%). Practices required a median (interquartile range) of 8.2 (4.6-11.9) months to report any ABCS eCQM. Time to report differed by eCQM: practices reported blood pressure management the fastest (median [interquartile range], 7.8 [3.5-10.4] months) and cholesterol management the slowest (median [interquartile range], 10.5 [6.6 to >12] months) (log-rank *P* < .001). In multivariable models, the blood pressure eCQM was reported more quickly by practices that participated in accountable care organizations (hazard ratio [HR], 1.88; 95% CI, 1.40-2.53; *P* < .001) or participated in a quality demonstration program (HR, 1.58; 95% CI, 1.14-2.18; *P* = .006). The cholesterol eCQM was reported more quickly by practices that used clinical guidelines for cardiovascular disease management (HR, 1.35; 95% CI, 1.18-1.53; *P* < .001). Compared with Federally Qualified Health Centers, hospital-owned practices had greater ability to report blood pressure eCQMs (HR, 2.66; 95% CI, 95% CI, 1.73-4.09; *P* < .001), and clinician-owned practices had less ability to report cholesterol eCQMs (HR, 0.52; 95% CI, 0.35-0.76; *P* < .001).

**Conclusions and Relevance:**

In this study, time to report eCQMs varied by measure and practice type, with very few practices reporting quickly. Practices took longer to report a new cholesterol measure than other measures. Programs that require eCQM reporting should consider the time and effort practices must exert to produce reports. Practices may benefit from additional support to succeed in new programs that require eCQM reporting.

## Key Points

**Question:**

How quickly can primary care practices report electronic clinical quality measures based on evidence-based guidelines for cardiac care?

**Findings:**

In this quality improvement study of 211 primary care practices, the median time to report any baseline electronic clinical quality measure was 8.2 months. Time to report varied by measure type and practice characteristics.

**Meaning:**

This study suggests that clinical quality measure reporting still takes a great deal of time and effort, and as the health care system increasingly moves to value-based structures that require electronic clinical quality measures, some practices may be left behind without better incentives and support.

## Teaser

This quality improvement study evaluates time to reporting electronic clinical quality measures for US primary care practices.

## Introduction

The Health Information Technology for Economic and Clinical Health (HITECH) Act, passed in 2009 as a part of the American Recovery and Reinvestment Act, specified general guidelines for the development and implementation of a “nationwide health information technology infrastructure.”^1^ Through HITECH Act initiatives, the federal government has spent significant time and money to promote widespread adoption of electronic health records (EHRs) that were intended to improve the quality, safety, efficiency, coordination, and equity of health care in the United States.^2,3^ Among other purposes, EHRs were to offer a standardized platform to better demonstrate gains in these domains. A key feature of the infrastructure was promotion of clinical quality with reporting measures that would be collected and reported using certified EHR systems.

The increasing prevalence of EHRs has prompted the electronic extraction of clinical quality measures (eCQMs) to become the standard for quality reporting programs. Reporting burden has grown over time, with increasing requirements to report eCQMs for a variety of quality initiatives^4^ and value-based reimbursement structures.^5,6^ This growing burden has major implications for resource allocation: estimates suggest that the time primary care team members spend on eCQM reporting equates to billions of dollars per year.^7^ Thousands of eCQMs have been developed by independent groups, with different ones used in various governmental or payer initiatives, leading to confusion and fatigue on the part of health care practices.^8^

Numerous barriers influence primary care practice teams’ ability to efficiently and accurately report eCQMs, including questionable data accuracy and variation in validity across measures and physicians.^9,10,11,12^ Furthermore, the extent to which eCQMs correspond to quality care and improved outcomes has been questioned.^13^ Variable data documentation practices greatly affect data completeness and reliability.^10,12^ Barriers to eCQM reporting and meaningful use of data include the time and effort required to implement reporting processes, resistance to change, limited EHR reporting functionality, costs, inflexible reporting criteria, inconsistency between measures and clinical guidelines, and vendors who were unreceptive to requests for flexible EHR configuration.^12,14^ Small practices may be more likely to experience financial barriers related to EHR adoption and use.^15^ Variation in definition of measures, data sources, and data formats may limit the comparability and utility of quality measures across practices.^13^

A number of efforts have aimed to reduce the burden of measure reporting on practices by increasing the adoption and meaningful use of health information technology, identifying and addressing gaps in primary care teams’ data skills, focusing on measures that matter,^16^ improving clarity of measure specifications, and aligning measures across settings and outcomes.^17^ Professional societies have supported the use of data analytics platforms like PRIME Registry.^18^ Other known facilitators of eCQM reporting,^12,19^ such as onsite training, local technical support, and opportunities for harmonization and shared learning, have been advanced by federal and state programs.^20,21,22^

The EvidenceNOW Southwest (ENSW) project offered an opportunity to see whether primary care practices have developed capacity to produce eCQMs. The ENSW project is a collaborative effort between Colorado and New Mexico covering the diverse geographic and cultural regions of both states. It is 1 of 7 regional cooperatives funded by the Agency for Healthcare Research and Quality’s (AHRQ) EvidenceNOW research study that started in 2015 to help small- and medium-sized primary care practices use the latest evidence to improve cardiovascular health. The ENSW project built upon the efforts described to reduce eCQM reporting burden by choosing a minimum number of measures known to prolong life and improve health, matching reporting specifications as closely as possible to these clinical standards, accepting a variety of data sources (eg, EHRs, patient-level extracts, third-party registries), and offering robust technical assistance through onsite clinical health information technology advisors, access to regional experts, and linkages to AHRQ’s national technical assistance contractor.

In this study, we sought to take advantage of the opportunity presented by the ENSW project’s use of 4 common and standardized eCQMs to examine how quickly primary care practices could report on these eCQMs. Our hypothesis was that, nearly 10 years following the HITECH Act, many primary care practices still do not possess the skills and tools to easily meet basic eCQM reporting requirements and that practices with certain characteristics experience greater delays than others when reporting eCQMs.

## Methods

Practice recruitment and selection for participation in ENSW has been described elsewhere.^23^ The ENSW project and the study described in this article were approved by the Colorado Multiple Institutional Review Board and the University of New Mexico Human Research Protections Office. The ENSW project is registered on ClinicalTrials.gov (NCT02515578). Participants completing surveys were provided written information about the study. The need to document consent was waived by the human subjects review boards because they determined that the research presented no more than minimal risk of harm to participants and involved no procedures for which written consent was required outside of the research context. All participants were provided with an informed consent document in the form of an information sheet explaining the research aims, patient rights, and potential risks. This report follows the Standards for Quality Improvement Reporting Excellence (SQUIRE) reporting guideline.^24^

### Measure Selection and Practice Support

The AHRQ selected the measures of aspirin use,^25^ blood pressure control,^26^ cholesterol management,^27^ and smoking cessation^28^ (ABCS) to advance heart health in alignment with the Million Hearts Campaign,^29^ the National Quality Forum, and the Centers for Medicare & Medicaid Services. The AHRQ selected standard eCQM specifications for use by all practices participating in the 7 cooperatives.^30^ These specifications included a 12-month measurement period for each quarterly report.

Recognizing potential challenges to eCQM reporting, in addition to receiving 9 months of ongoing practice transformation support from a trained practice facilitator, ENSW provided practices with support from a clinical health information technology advisor and resources and support from the research team, which had experience collecting eCQMs. This support team assisted practices with developing and managing workflows for data collection, reporting, and analysis; helped with the entry of eCQMs into the reporting website; and linked practices with other technical assistance resources as needed and available. Practices were instructed to report their baseline ABCS eCQMs as soon as possible once their practice transformation support began.

### eCQM Reporting Mechanisms

The ENSW project offered practices several options to report eCQM data to a centralized repository. The first option allowed practices to calculate eCQM numerators and denominators using an internal EHR or registry. These data were manually entered through an online portal. The other option allowed practices to securely transfer patient-level information to the DARTNet Institute^31^ through structured flat files or direct EHR data extraction. The DARTNet Institute then normalized the clinical data, calculated the eCQMs, and reported results on the practice’s behalf.

### Practice Characteristics and Context

Practice characteristics were obtained from the baseline ENSW Practice Survey. At least 1 staff member, typically a practice administrator or lead clinician, completed the Practice Survey for each participating practice. The baseline Practice Survey gathered descriptive information on participating practices, including a series of questions surrounding use of strategies for improving patient care, such as quality improvement processes and patient self-management support.

Practice ownership was consolidated for these analyses to 3 categories: (1) clinician (including solo or group practices); (2) hospitals and academic centers (including academic health centers, faculty practices, hospital or health system practices, or health maintenance organizations); and (3) Federally Qualified Health Centers (FQHC) (including FQHCs, FQHC lookalike clinics, and Rural Health Clinics). Practice size was defined as the number of clinicians working at that site. Practice zip code aligned to Rural-Urban Commuting Area codes was used to determine geographic area. We assigned practices with zip codes corresponding with Rural-Urban Commuting Area codes 1 to 4 as rural and 5 to 10 as urban or suburban.^32^ Cardiovascular disease (CVD) registries included a count of the following registries in use at the practice: ischemic vascular disease, hypertension, high cholesterol, diabetes, prevention services, and registries for high-risk patients. Total number of registries was translated into an ordered categorical variable (0, 1-2, 3-4, and 5-6). A score for adoption of CVD guidelines for prevention and management was created by counting the following activities reported by a practice: guidelines posted or distributed, clinicians’ agreed-on guidelines, standing orders created, or EHR prompts for each type of guideline. Accountable care organization (ACO) member options included Medicaid, Medicare, private or commercial, and other.

The survey also asked about major practice changes, including using a new or different EHR, moving to a new location, losing 1 or more clinicians, losing the office manager or head nurse, being purchased by or affiliating with a larger organization, or implementing a new billing system. Practices were asked if they previously participated in payment or quality demonstration programs including a State Innovation Model initiative, Comprehensive Primary Care Initiative, Transforming Clinical Practice Initiative, Community Health Worker training program, Blue Cross/Blue Shield Patient-Centered Medical Home program, Million Hearts State Learning Collaborative, Million Hearts Cardiovascular Disease Risk Reduction Model, or other program. Previous quality reporting support options included receiving help from any health information exchange, practice-based research network, clinical data warehouse, external consulting group, health system practice network, hospital network, primary care association, or regional extension center. Practice incentive or bonus payment options included the Medicare primary care incentive payment or the Medicare care coordination payment.

### Time to Report

The primary outcome measure for our analyses was time to reporting. We calculated time to report as a measurement of time in days (converted to months to aid interpretability) from the date of the practice’s kickoff meeting with ENSW transformation support staff to submission of baseline eCQM data for each of the ABCS measures. Using the ENSW kickoff date ensured a discretely recorded, objective time zero uniformly available for all practices in the study.

### Statistical Analysis

Descriptive statistics were generated for practice characteristics (eg, frequencies, proportions, mean, standard deviation). The outcome variables for all analyses are time to reporting for each eCQM and time to reporting for the first eCQM reported. Practices that had not reported by the end of the assessment period for this analysis (November 1, 2017) were censored as of that date. Practices had a minimum of 7.7 months from the time the practice first received transformation support to the end of the assessment period. The log-rank test was used to generate product-limit curves and compare survival distributions across the measures. For blood pressure and cholesterol, Cox proportional hazards regression models were used to examine practice characteristics that were associated with time to reporting in univariable and multivariable models. Practices that dropped out immediately after the kickoff meeting were excluded from analysis (n = 6); practices that dropped out more than 1 month after kickoff and had not reported measures (n = 3) were censored at the time of dropout. Backward elimination was used to arrive at the final multivariate models, initially including all variables that were significant at *P* < .10 and eliminating variables 1 at a time until all were *P* < .05.^33^ The threshold for statistical significance of results was *P* < .05 using 2-sided tests. Because of the variable length of assessment periods for practices, sensitivity analyses were performed limiting the observation period to a maximum of 12 months to determine whether there was bias associated with longer observation time for some practices enrolled earlier. All analyses were performed using SAS statistical software version 9.4 (SAS Institute Inc).

## Results

Data represent 211 enrolled practices that provided survey and eCQM data between January 1, 2015, and November 1, 2017. Table 1 details the characteristics of participating practices. Most practices (75%) were in Colorado. Practices were predominantly clinician owned (48%), located in urban or suburban areas (71%), and used at least 1 patient registry (68%) at baseline. The mean (SD) practice size was 3.5 (2.6) clinicians. Approximately 47% of practices reported participating in some type of ACO. A substantial majority (85%) calculated eCQMs using their EHR or internal registry.

**Table 1.** Practice Characteristics

Characteristic		Practices, No. (%)^a^
Total practices		211
Ownership		
Clinician		101 (47.9)
Hospital or academic center		33 (15.6)
Federally Qualified Health Centers or Rural Health Clinic		77 (36.5)
Practice size, mean (SD) No. of clinicians (n = 206)		3.5 (2.6)
Geographic area		
Rural		61 (28.9)
Urban or suburban		150 (71.1)
Cardiovascular disease registries		
Ischemic vascular disease		80 (37.9)
Hypertension		114 (54.0)
Diabetes		135 (64.0)
High cholesterol		94 (44.6)
No. of cardiovascular disease registries, mean (SD) (n = 211)^b^		2.89 (2.38)
Adoption of cardiovascular disease clinical guidelines		
No. of prevention guidelines, mean (SD) (n = 211)		1.82 (1.37)
No. of management guidelines, mean (SD) (n = 211)		1.74 (1.37)
Accountable care organization member		100 (47.4)
Patient-Centered Medical Home recognized		94 (44.6)
Using Meaningful Use–certified electronic health record		191 (93.6)
Participated in Meaningful Use stage 1		143 (67.8)
Participation in comprehensive primary care initiative		11 (5.2)
≥2 Major changes in practice		49 (23.2)
Participation in any payment or quality demonstration program		62 (29.4)
Previous quality reporting support		138 (65.4)
Practice incentive payments		
Medicare care coordination		11 (5.2)
Medicare primary care		47 (22.3)
Electronic clinical quality measure reporting mechanism		
Internal electronic health record or registry		179 (84.8)
External electronic clinical quality measure tool (DARTNet Institute)		18 (8.5)
Unable to report any electronic clinical quality measure		14 (6.6)
Electronic health record		
Allscripts		4 (1.9)
Amazing Charts		9 (4.3)
Athena Health		10 (4.7)
Cerner		4 (1.9)
eClinicalWorks		22 (10.4)
e-MDs		17 (8.1)
EPIC		22 (10.4)
GE/Centricity		6 (2.8)
Greenway Medical		19 (9)
McKesson/Practice Partner		2 (1)
NexGen		59 (28)
Practice Fusion		6 (2.8)
Other		24 (11.4)

^a^ Percentages might not sum to 100 because of missing data; not all practices responded to all questions because of skip logic in the survey.

^b^ Number of cardiovascular disease registries included a count of the following registries in use at the practice: ischemic vascular disease, hypertension, high cholesterol, diabetes mellitus, prevention services, and registries for high risk patients. A score for adoption of cardiovascular disease guidelines for prevention and management was created by counting the following activities reported by a practice: guidelines posted or distributed, clinicians agreed on guidelines, standing orders created, or electronic health record prompts for each type of guideline.

### Time to Report

The median (interquartile range [IQR]) time to report any measure was 8.2 (4.6-11.9) months. The median (IQR) time to report varied across measures from a minimum of 7.8 (3.5-10.4) months for the blood pressure measure to a maximum of 10.5 (6.6 to >12) months for the cholesterol measure (Table 2). Few practices reported measures within 6 months, ranging from a minimum of 22.8% of practices for the cholesterol measure to a maximum of 34.6% of practices for the blood pressure measure.

**Table 2.** Time to Report Aspirin Prescribing, Blood Pressure Control, Cholesterol Management, and Smoking Cessation Electronic Clinical Quality Measures

Measure	Time to Report, Median (IQR), mo^a^	Practices Reporting by 6 mo, No. (%)
Blood pressure management	7.8 (3.5-10.4)	73 (34.6)
Aspirin	8.1 (4.6-10.9)	59 (28.0)
Smoking cessation	8.2 (4.5-10.8)	59 (28.0)
Cholesterol management	10.5 (6.6 to >12)	48 (22.8)

Abbreviation: IQR, interquartile range.

^a^ Time was measured from when a practice first started to receive transformation support.

The Figure plots the proportion of practices reporting each eCQM over time. Sensitivity analyses limited the maximum observation time frame to 12 months. Practices demonstrated a lower probability of reporting the cholesterol measure within the 12-month observation period (log-rank test for equality over strata: χ^2^_3_ = 41.42; *P* < .001).

**Figure.**
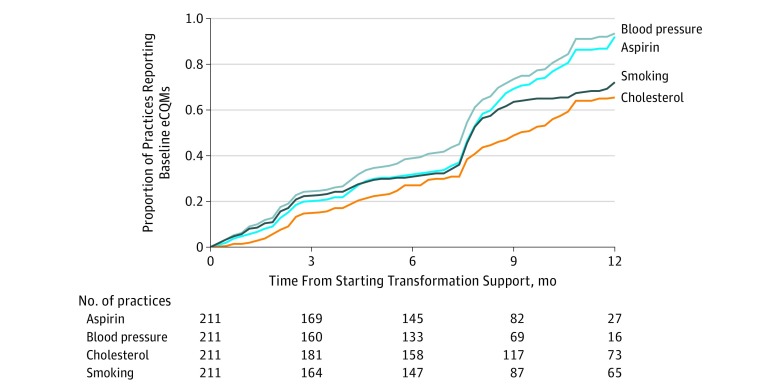
Time for Practices to Report Different Electronic Clinical Quality Measures (eCQMs) The median (interquartile range) time to report any measure was 8.2 (4.6-11.9) months, with a minimum of 7.8 months for the blood pressure measure to a maximum of 10.5 months for the cholesterol measure.

Practices that used the DARTNet Institute reported the cholesterol measure faster (median [IQR] time to report, 7.0 [4.5-9.7] months) than practices using their own EHR (median [IQR] time to report, 8.9 [5.7-14.8] months) (log-rank *P* = .004). The times to report the 3 other measures were not significantly different.

Practices in the study used more than 13 different EHRs encompassing 48 different EHR versions, with the most common EHR, NextGen, used by 28% of practices. Because of the relatively small number of practices using any particular EHR, we did not assess these in Cox regression models. We provide median time to report for EHRs used by more than 5 practices in the eTable in the Supplement.

### Practice Characteristics Associated With Ability to Report Certain eCQMs

Hazard ratios (HRs) from the univariable Cox proportional hazards models are shown in Table 3 for the blood pressure and cholesterol measures. Results for aspirin and smoking cessation measures are not presented because the patterns were similar to blood pressure results.

**Table 3.** Univariable Analyses of Practice Characteristics Associated With Less Time to Report Certain Electronic Clinical Quality Measures

Measure	Unadjusted HR (95% CI) (N = 205)^a^	
	Blood Pressure Management	Cholesterol Management
Ownership		
Clinician	1.42 (1.04-1.93)^b^	0.41 (0.29-0.60)^b^
Hospital or academic center	2.41 (1.58-3.66)^b^	1.08 (0.69-1.70)
Federally Qualified Health Center or Rural Health Clinic	1 [Reference]	1 [Reference]
Practice size, No. of clinicians	1.06 (1.01-1.12)^b^	1.04 (0.98-1.10)
Accountable care organization member	1.94 (1.44-2.61)^b^	1.37 (1.00-1.88)
Greater use of patient registries, ordinal^c^	0.98 (0.88-1.09)	1.23 (1.08-1.39)^b^
Adoption of clinical guidelines for cardiovascular disease prevention	1.09 (0.98-1.22)	1.40 (1.24-1.58)^b^
Adoption clinical guidelines for cardiovascular disease management	1.12 (1.01-1.26)^b^	1.41 (1.25-1.59)^b^
Patient-Centered Medical Home recognized	0.72 (0.54-0.96)^b^	1.13 (0.82-1.55)
Previous quality reporting support	1.35 (1.001-1.82)^b^	1. 58 (1.11-2.23)^b^
Participation in any payment or quality demonstration program	1.46 (1.07-2.00)^b^	0.95 (0.67-1.35)
Practice incentive payments		
Medicare care coordination	1.41 (0.74-2.68)	0.75 (0.33-1.70)
Medicare primary care	0.90 (0.64-1.26)	0.50 (0.32-0.76)

Abbreviation: HR, hazard ratio.

^a^ Unadjusted HRs shown for any variable with *P* < .25 with 95% confidence limit (risk limits).

^b^ Statistically significant at *P* < .05.

^c^ Cardiovascular disease registries included a count of the following registries in use at the practice: ischemic vascular disease, hypertension, high cholesterol, diabetes, prevention services, and registries for high-risk patients. A score for adoption of cardiovascular disease guidelines for prevention and management was created by counting the following activities reported by a practice: guidelines posted or distributed, clinicians agreed on guidelines, standing orders created, or electronic health record prompts for each type of guideline.

Practice characteristics associated with greater ability to report eCQMs varied between the blood pressure and cholesterol measures. Earlier ability to report the blood pressure measure was associated with ownership by clinicians (HR, 1.42; 95% CI, 1.04-1.93) or hospitals (HR, 2.41; 95% CI, 1.58-3.66) vs FQHC, larger size (HR, 1.06; 95% CI, 1.01-1.12), ACO participation (HR, 1.94; 95% CI, 1.44-2.61), greater use of clinical guidelines for CVD management (HR, 1.12; 95% CI, 1.01-1.26), previous quality reporting support (HR, 1.35; 95% CI, 1.001-1.82), and participation in a payment or quality demonstration program (HR, 1.46; 95% CI, 1.07-2.00). Patient-Centered Medical Home recognition was associated with less ability to report the blood pressure eCQM (HR, 0.72; 95% CI, 0.54-0.96). For the cholesterol measure, practice characteristics associated with greater ability to report included being an FQHC vs clinician owned (HR for clinician owned, 0.41; 95% CI, 0.29-0.60), greater use of patient registries (HR, 1.23; 95% CI, 1.08-1.39), greater use of clinical guidelines for CVD prevention and management (HR for prevention, 1.40; 95% CI, 1.24-1.58 and HR for management, 1.41; 95% CI, 1.25-1.59), and participation in a payment or quality demonstration program (HR, 1.46; 95% CI, 1.07-2.00). Receiving incentive payments for Medicare primary care was associated with less ability to report cholesterol eCQMs (HR, 0.75; 95% CI, 0.33-0.76).

Multivariate models indicated that ACO participation (HR, 1.88; 95% CI, 1.40-2.53; *P* < .001), hospital ownership vs FQHC (HR, 2.66; 95% CI, 1.73-4.09; *P* < .001), and participation in a payment or quality demonstration (HR, 1.58; 95% CI, 1.14-2.18; *P* = .006) were associated with greater ability to report blood pressure management (Table 4). For cholesterol measure reporting, FQHC (vs clinician-owned) practices (HR for clinician ownership, 0.52; 95% CI, 0.35-0.76; *P* < .001) and greater use of clinical guidelines for CVD management (HR, 1.35; 95% CI, 1.18-1.53; *P* < .001) were associated with greater ability to report. Results were very similar in sensitivity analyses limiting the maximum time to 12 months (but with slightly less power).

**Table 4.** Final Multivariable Models of Select Practice Characteristics Associated With Ability to Report Electronic Clinical Quality Measures

Characteristic	Blood Pressure Management		Cholesterol Management	
	HR (95% CI)^a^	*P* Value	HR (95% CI)^a^	*P* Value
Ownership				
Clinician	1.31 (0.96-1.78)	<.001	0.52 (0.35-0.76)	<.001
Hospital or academic center	2.66 (1.73-4.09)		1.41 (0.89-2.26)	
Federally Qualified Health Centers or Rural Health Clinic	1 [Reference]		1 [Reference]	
Any accountable care organization participation	1.88 (1.40-2.53)	<.001	NA	NA
Use of clinical guidelines for cardiovascular disease management	NA	NA	1.35 (1.18-1.53)	<.001
Payment or quality demonstration programs, any	1.58 (1.14-2.18)	.006	NA	NA

Abbreviations: HR, hazard ratio; NA, not applicable.

^a^ Final multivariable models show Cox proportional hazards regression of select practice characteristics associated with ability to report electronic clinical quality measures.

## Discussion

Our study sought to examine the current capacity of primary care practices to report 4 evidence-based eCQMs. Despite nearly all participating practices using Meaningful Use–certified EHRs and the provision of dedicated health information technology support by the ENSW project, primary care practices still required a substantial amount of time and support to report even well-established eCQMs. The ability to report ABCS eCQMs varied by measure type and practice characteristics.

Our results highlight how introducing new measures increases the reporting burden on practices. All 4 measures reflected current clinical care guidelines, but the aspirin, blood pressure management, and tobacco cessation measures were more established. Their specifications had been relatively stable, and they have been used for Centers for Medicare & Medicaid Services and other quality and research programs for many years. On the other hand, the cholesterol measure was chosen to reflect a very recent update to clinical guidelines. At the start of the ENSW project, there were no nationally recognized eCQM specifications for calculating the measure. Meaningful Use certification standards did not require the measure, and no payment program used the metric. Compared with blood pressure management, the new cholesterol measure took nearly 3 months longer for the typical practice to report (7.8 months vs 10.5 months, respectively).

Our results also show that certain types of practices are more capable of prompt eCQM reporting. Practices that participate in ACOs and systematically use clinical guidelines seem better prepared to report established measures like the blood pressure measure and new measures like the cholesterol measure. Hospital-owned practices more quickly reported the established measures, and FQHCs more quickly reported the new cholesterol measure. While these associations do not imply causation, it would stand to reason that some combination of a practice’s skill, previous activity, payment structures, and internal and external resources are leading to the variation in the time to reporting we observed.

Initial implementation of an EHR system has high costs in terms of time, training, finances, and lost productivity.^34,35,36^ Our findings indicate that these barriers do not end once EHR implementation is complete. For the practices we studied, substantial delays often continued well after the initial attempt to report measures. We found that it can take several months for a practice to produce any 1 of 4 standard measures. Implementing a new measure not previously adopted by federal programs like Meaningful Use takes practices even more time. We found a considerable time burden that health care teams face in reporting clinical quality measures, which builds on the previously reported estimate that physicians and their staff spend an average of 15 hours per week developing, collecting, and reporting external quality measures.^7^

Our findings agree with others’ conclusions that programs should try to align the amount and forms of health information technology support to best match practices’ needs.^37^ This article complements the work of Cohen et al,^14^ which looked at 1492 practices across the national EvidenceNOW project. Those practices reported on their ability to report on eCQMs at the outset of the project and the potential barriers to their use of EHR data for quality purposes. Our article adds to their findings by detailing the actual time to reporting for more than 200 participating practices. Our results are consistent with previous research demonstrating modest but inconsistent associations between select structural elements of primary care practices and performance on various quality measures.^38^ Practice size was positively associated with ability to report the blood pressure eCQM, which aligns with evidence that smaller practices may experience greater barriers and delays in EHR use than larger practices,^39,40^ perhaps suggesting that this disparity extends to the ability to report certain measures. Our findings also complement evidence that small practices need sustained and extensive EHR support to achieve improvement in quality measures.^41^ Beyond these studies, existing literature contains little information regarding the influence of contextual details with the use of health information technology.^42^

Barriers to meaningfully implementing EHRs and using EHR data are manifold: costs, lack of knowledge of EHR functions, problems transforming office operations, lack of standardization, vendor system upgrades, lack of dedicated data coordinators, staff and clinician resistance, and fatigue.^12,43,44^ Reliably reporting individual measures may be further influenced by the interplay—and unpredictability—of multiple factors: that is, the “complexity of the sociotechnical networks at stake.”^45^ Primary care practices are complex adaptive systems,^46,47^ and while our findings help identify specific practice characteristics that may be associated with quality measure reporting and performance, how these characteristics interact in any given practice is affected by the local landscape and factors beyond our ability to measure in this study. Practices using EHR data to inform quality improvement need ongoing and tailored support that can assist with addressing these complex factors.^48^

### Limitations

This study has several important limitations. Health information technology adoption can vary across regions and practice types,^49,50,51,52^ so generalizability beyond these small- to medium-sized primary care practices in the Southwest United States may be limited. Numerous unmeasured factors may have influenced time to report, including degree of leadership engagement in ENSW, the costs to create reports in the different eCQM production tools, competing demands, and actual time spent trying to produce eCQMs. Measures produced with internal EHRs or registries were not independently verified for accuracy beyond basic validation checks (eg, numerator must be less than or equal to denominator), and many practices further refined data collection workflows and eCQM calculation processes after reporting baseline eCQMs. Reporting valid, trusted, and actionable eCQMs takes even more time and effort. The ENSW project provided practices with a significant amount of technical support to facilitate eCQM reporting, including individualized help from a clinical health information technology advisor, peer learning networks, online measurement guides, and access to technical assistance. Programs that provide less support would likely encounter greater delays in eCQM reporting.

## Conclusions

Nearly a decade has passed since the HITECH Act was enacted, and our project that focused on small- to medium-sized practices highlights a success and a failure of that policy. Nearly all of the practices used Meaningful Use–certified EHRs. That is a major success. However, the inability to use those EHRs to quickly track and report on quality is a major failure. The ability to readily access and report trustworthy eCQM data has become an essential competency of primary care practice teams. Beyond the external reporting requirements, practices’ ability to use quality data to monitor and improve their performance is essential. Despite years of on-the-ground and systems-level work, our experience shows that eCQM reporting still takes a great deal of time and effort. As the health care system increasingly moves to value-based structures that require eCQMs, some practices may be left behind without better incentives and support. Health care leaders, policy makers, EHR vendors and technical assistance providers should continue their efforts to reduce the burden of eCQM reporting and improve data capacity in primary care practices.

## Notes

Supplement. **eTable.** Median Time, in Months, to Report ABCS Electronic Clinical Quality Measures by Electronic Health Record
