# Supplementary material for: Primary Care Practices’ Ability to Report Electronic Clinical Quality Measures in the EvidenceNOW Southwest Initiative to Improve Heart Health
Source: JAMA Netw Open. 2019 Aug 7;2(8):e198569. doi: 10.1001/jamanetworkopen.2019.8569 (PMC6687038; doi:10.1001/jamanetworkopen.2019.8569)
Supplement: Supplement. — eTable. Median Time, in Months, to Report ABCS Electronic Clinical Quality Measures by Electronic Health Record [file jamanetwopen-2-e198569-s001.pdf]

Supplementary Online Content

Knierim KE, Hall TL, Dickinson LM, et al. Primary care practices' ability to report electronic clinical quality measures in the EvidenceNOW Southwest initiative to improve heart health. *JAMA Netw Open*. 2019;2(8):e198569. doi:10.1001/jamanetworkopen.2019.8569

eTable. Median Time, in Months, to Report ABCS Electronic Clinical Quality Measures by Electronic Health Record

This supplementary material has been provided by the authors to give readers additional information about their work.

© 2019 Knierim KE et al. *JAMA Network Open.*

Supplement eTable: Median time, in months, to report ABCS electronic clinical quality measures by electronic health record.

	Blood Pressure Management	Aspirin	Smoking Cessation	Cholesterol Management
**Overall**	7.8	8.1	8.2	10.5
**Amazing Charts (n=9)**	7.13	7.13	8.97	4.31
**Athena Health (n=10)**	8.23	8.23	8.23	8.65
**eClinicalWorks (n=22)**	7.48	7.63	7.77	9.14
**e-MDs (n=17)**	4.47	4.47	7.73	14.83
**EPIC (n=22)**	2.55	2.55	2.55	2.55
**GE/Centricity (n=6)**	7.82	7.82	7.82	7.82
**Greenway Medical (n=19)**	4.60	4.60	4.80	8.88
**NextGen (n=59)**	8.09	8.84	12.26	8.05
**Practice Fusion (n=6)**	7.38	7.38	7.38	8.09
**Other* (n=34)**	6.44	8.45	7.73	17.72

Supplement eTable. Median time, in months, is measured from when a practice first started to receive transformation support from the project. *Includes electronic health record systems used by 5 or fewer practices

© 2019 Knierim KE et al. *JAMA Network Open.*
